# Low-acrylamide French fries and potato chips

**DOI:** 10.1111/j.1467-7652.2008.00363.x

**Published:** 2008-10

**Authors:** Caius M Rommens, Hua Yan, Kathy Swords, Craig Richael, Jingsong Ye

**Affiliations:** Simplot Plant Sciences, J. R. Simplot CompanyBoise, ID 83706, USA

**Keywords:** acrylamide, intragenic, plant biotechnology, potato

## Abstract

Acrylamide is produced in starchy foods that are baked, roasted or fried at high temperatures. Concerns about the potential health issues associated with the dietary intake of this reactive compound led us to reduce the accumulation of asparagine, one of its main precursors, in the tubers of potato (*Solanum tuberosum*). This metabolic change was accomplished by silencing two asparagine synthetase genes through ‘all-native DNA’ transformation. Glasshouse-grown tubers of the transformed intragenic plants contained up to 20-fold reduced levels of free asparagine. This metabolic change coincided with a small increase in the formation of glutamine and did not affect tuber shape or yield. Heat-processed products derived from the low-asparagine tubers were also indistinguishable from their untransformed counterparts in terms of sensory characteristics. However, both French fries and potato chips accumulated as little as 5% of the acrylamide present in wild-type controls. Given the important role of processed potato products in the modern Western diet, a replacement of current varieties with intragenic potatoes could reduce the average daily intake of acrylamide by almost one-third.

## Introduction

Asparagine plays an apparently important role in the assimilation and storage of nitrogen (Lam *et al*., 2003), and is particularly abundant in the products of wheat (*Triticum aestivum*) (Benedito *et al*., 1989), coffee (*Coffea arabica* and *C. canephora*) (Mazzafera, 1999) and potato (Talley *et al*., 1970). On heat processing, the amide amino acid reacts with reducing sugars to produce acrylamide (Mottram *et al*., 2002). Average daily intake levels of this Maillard reaction product are estimated to be 0.3–0.7 µg acrylamide/kg/day (Dybing *et al*., 2005). Ingested acrylamide is readily absorbed and metabolized, in part, by a cytochrome P450 to produce mercapturic acid and glycidamide (Boettcher *et al*., 2005). Although mercapturic acid is excreted via the urine, both the remaining acrylamide and its reactive metabolite bind to various proteins as well as DNA (Barber *et al*., 2007; Martins *et al*., 2007). High levels of adduct formation have been linked to animal health issues, including cumulative nerve terminal damage (Johnson *et al*., 1986; Friedman *et al*., 1995). In humans, oral intake levels believed to be without an appreciable risk of deleterious effects are currently estimated to be 3.0 µg acrylamide/day (http://www.epa.gov/iris). This level of dietary intake is exceeded in small subsets of the population, particularly in young children and adolescents (Dybing *et al*., 2005). In their preliminary JECFA/64/SC report (http://www.who.int/ipcs/food/jecfa/summaries/summary_report_64_final.pdf), the Joint Food and Agriculture Organization/World Health Organization (FAO/WHO) Expert Committee on Food Additives and Contaminants has therefore recommended reducing the acrylamide content of processed starchy foods.

Recently developed methods to limit acrylamide formation require changes in grower or processor practices, which may limit their broad application. For instance, the beneficial effect of sulphur fertilization on lowering the acrylamide potential of potato and wheat (Elmore *et al*., 2007) is offset by increased farmer input costs and sulphur contamination issues. Furthermore, the partial decreases in acrylamide concentration that can be achieved by modifying processing variables, such as the time and temperature of heating (Rydberg *et al*., 2005; Amrein *et al*., 2006), yield products that have lost some of their original colour, flavor and/or texture, and may therefore be less appealing to consumers. A third approach incubates raw materials with either asparagine-metabolizing enzymes or amino acids that compete with asparagine in the Maillard reaction (Elder *et al*., 2006; Howie *et al*., 2006). Such treatments have been shown to be only partially effective for some raw food ingredients, require high concentrations of the additive, and are too difficult and costly to apply broadly. A preferred route to the reduction of acrylamide would be to shift to crops that are naturally poor in acrylamide precursors. However, there are currently no such varieties available that also display all the additional input, processing and quality traits demanded by the processing industry (Vivanti *et al*., 2006). Given the complexity of wheat, coffee and potato breeding, efforts to develop such new processing varieties will require 15 years or more.

A faster route to decrease the acrylamide potential of food crops was established 2 years ago through genetic engineering. Simultaneous silencing of two tuber-expressed genes in starch degradation, which encode water dikinase R1 and amyloplast-targeted phosphorylase-L, led to a decrease in the accumulation of glucose and fructose by approximately twofold (Rommens *et al*., 2006). Reduced browning of processed products from these modified tubers correlated with an approximately two- to threefold decrease in acrylamide levels. In this article, an alternative approach for the production of low-acrylamide French fries and potato chips is described that does not alter their sensory characteristics. This new method is based on the tuber-specific silencing of two genes in asparagine biosynthesis, and reduces the concentration of free asparagine by up to 95%.

## Results

### Silencing of asparagine synthase genes limits asparagine biosynthesis in potato tubers

**Figure 1:**
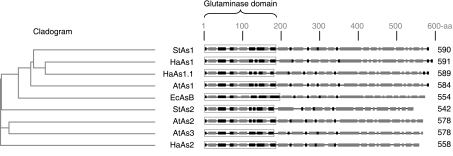
Phylogenetic relationship and structure of asparagine synthetase (As) proteins from a number of different species: At, *Arabidopsis thaliana*; Ec, *Escherichia coli*; Ha, *Helianthus annuus*; St, *Solanum tuberosum*. Black boxes, α helices; grey boxes, β sheets.

Asparagine synthetase (As) catalyses the ATP-dependent conversion of aspartate into asparagine. Two cDNAs for this key enzyme in asparagine biosynthesis, designated as *StAs1* and *StAs2*, were amplified from a tuber poly(A)^+^ mRNA-derived library of the potato variety Ranger Russet. The 1.6-kb open reading frames share 71% sequence identity and encode products with a typical ~190-amino-acid N-terminal glutaminase domain (cd00712), which is involved in the hydrolysis of glutamine to produce glutamate and ammonia (Marchler-Bauer *et al*., 2007) (Figure 1). The predicted secondary structures of both of these proteins and their functional orthologues resemble that of the crystallized AsB of *Escherichia coli* (Larsen *et al*., 1999) (Figure 1). StAs1 shares most homology with the proteins encoded by the dark-inducible *HaAs1* from sunflower (*Helianthus annuus*) (86%) and the *AtAs1* gene from *Arabidopsis* (83%). In contrast, StAs2 is more closely related to the product of the weakly expressed and light-inducible *AtAs2* gene (86%) (Figure 1).

**Figure 2:**
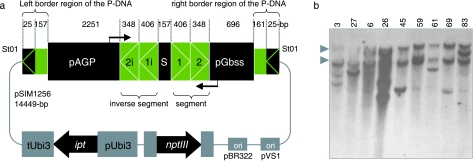
Plant transformation with a silencing construct targeting the potato asparagine synthetase genes (*StAs1* and *StAs2*) in tubers. (a) Diagram of vector pSIM1256. St01, potato-derived border-like element resembling the T-DNA border; pAGP, promoter of the potato *Agpase* gene; 1, *StAs1* gene fragment; 2, *StAs2* gene fragment; i, inverse complement; S, spacer; pGbss, promoter of the potato *Gbss* gene; ori, origin of replication; pUbi3, promoter of the potato *Ubi3* gene; *ipt*, Agrobacterium *ipt* gene; tUbi3, terminator of the potato *Ubi3* gene; *npt III*, kananycin resistance gene from *E. coli*. Plant-derived sequences are shown in green; vector backbone sequences are shown in grey. (b) P-DNA copy number of nine intragenic lines, as determined with an *StAs1/2*-derived probe that also visualizes two endogenous fragments (grey arrows).

In an attempt to limit the accumulation of asparagine in potato tubers, a silencing construct was designed that simultaneously targets the expression of *StAs1* and *StAs2* (Figure 2a). The two genes are quite divergent at the DNA level, with short (approximately 5 bp) stretches of homology interrupted by mismatches. Therefore, 0.4-kb fragments from both *StAs1* and *StAs2* were fused to create a DNA segment, and two copies of this segment were inserted as an inverted repeat between two convergently orientated promoters. It was our primary intent to reduce the transcription of the *StAs1* and *StAs2* genes in tubers, whilst limiting the extent of gene silencing in the foliage where the gene products play important roles in both photorespiration (Ta *et al*., 1985) and ammonium detoxification (Wong *et al*., 2004). For this reason, promoters were employed that were at least 100-fold more active in tubers than in leaves: the granule-bound starch synthase (Gbss) and a short (2.2-kb) version of the potato ADP-glucose pyrophosphorylase (Agp) (Visser *et al*., 1991; Muller-Rober *et al*., 1994; C. Richael, unpubl. data). The resulting silencing construct was positioned within a potato-derived transfer (P-) DNA to create the binary vector pSIM1256.

**Figure 3:**
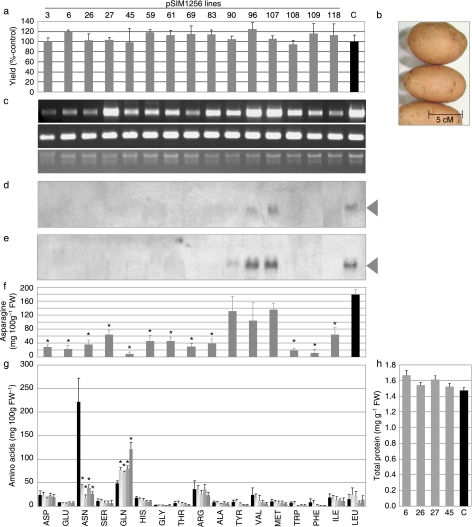
Silencing of the potato asparagine synthetase genes (*StAs1* and *StAs2*) decreases the accumulation of asparagine. (a) Tuber yield for 3-month-old glasshouse-grown plants of 15 intragenic lines (grey bars) and untransformed controls (C) (black bar). 100% = 361 ± 60 g/plant. (b) Phenotype of typical tubers of line 1256-26. (c) Semi-quantitative reverse transcriptase polymerase chain reaction (RT-PCR) for relative expression of *StAs2* (top panel) and the internal *actin* gene control (middle panel), with RNA loading shown in the bottom panel. (d) StAs2 transcript levels determined by RNA gel blot analysis. The grey arrow shows the predicted position of the RNA. Weak band intensity in the control lane indicates low *As2* gene expression levels in untransformed plants. Transcript levels were undetectable in tubers of most intragenic lines. (e) Hybridization of an RNA gel blot with an *StAs1* gene-derived probe. (f) Tuber asparagine levels. Significant differences from controls (*P* < 0.05) are indicated with an asterisk. FW, fresh weight. (g) Amino acid profiles for untransformed control tubers (black bars) and tubers from lines 1256-6 (lightest grey bar), 1256-26 (light grey bar), 1256-27 (grey bar) and 1256-45 (dark grey bar). (h) Total protein content. Data represent the mean of three experiments ± standard deviation.

An *Agrobacterium* LBA4404 strain containing pSIM1256 was used for marker-free and all-native DNA transformation of the ‘French fry’ variety Ranger Russet (Richael *et al*., 2008). Fifteen shoots with a P-DNA^+^/backbone^−^ genotype were rooted, confirmed by DNA gel blot analysis to contain at least one P-DNA integration event (Figure 2b, and data not shown) and propagated *in vitro* for the production of intragenic lines. Three plants of each of the 15 transformed lines were grown in the glasshouse for 3 months. Both the yield and size of harvested tubers were similar to those of untransformed controls (Figure 3a,b). The specific gravity, which is a reliable indicator of starch content, was also unaffected by the genetic modification (data not shown). An association between the presence of the silencing construct and two- to fivefold decreased transcript levels for the *StAs2* gene in tubers of 12 of the 15 lines was subsequently demonstrated by reverse transcriptase polymerase chain reaction (RT-PCR) (Figure 3c). RNA gel blot analysis, although less sensitive, confirmed this result (Figure 3d), and also indicated that silencing of the *StAs2* gene was fully correlated with suppressed expression of the *StAs1* gene (Figure 3e). The effective establishment of *StAs1/2* gene silencing in intragenic lines was mainly attributed to the presence of fragments from the two targeted genes in the silencing construct. In addition, it is possible that the partial homology between the *StAs1* gene fragment and the resident *StAs2* gene, and vice versa, was sufficient for some cross-silencing (Jackson *et al*., 2003).

Tubers from all the intragenic lines were analysed by high-performance liquid chromatography (HPLC) to determine the effect of *StAs1/2* gene silencing on the accumulation of asparagine. As shown in Figure 3f, the content of this amide amino acid was up to 20-fold lower in intragenic tubers than in the tubers transformed with an ‘empty’ vector control. Interestingly, tubers of line 1256-45 contained higher As2 transcript levels but lower concentrations of asparagine than tubers of, for instance, line 1256-26. This finding suggests that the various intragenic lines differ in their content of reducing sugars and/or other compounds that influence the potential for acrylamide formation (Rydberg *et al*., 2005). Further studies on four randomly chosen lines, 1256-6, 1256-26, 1256-27 and 1256-45, demonstrated that reduced asparagine levels coincided with a 1.5–2.5-fold increase in the accumulation of the alternative amide amino acid glutamine, which resembles asparagine in that it contains two amino groups (Figure 3g). All other tested amino acids remained at wild-type levels (Figure 3g), and the protein content was also unaltered (Figure 3h). However, the total amount of tested free amino acids in tubers from line 1256-26, which contained the smallest amount of asparagine, was 16% less than that of controls and other intragenic lines, indicating that an almost complete impairment of asparagine formation may have a rippling negative effect on the biosynthesis of other amino acids.

**Figure 4:**
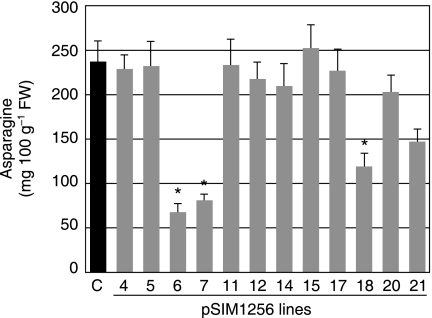
Asparagine levels of tubers from pSIM1151 lines. The results shown represent the average values of three measurements ± standard deviation for tubers of the untransformed control (C) (black bar) and 12 transformed lines (grey bars). Significant differences from controls (*P* < 0.05) are indicated with an asterisk. FW, fresh weight.

A second expression cassette that contained the *StAs1/2*-derived inverted repeat between two Gbss promoters was also tested for its efficacy to reduce the accumulation of asparagine in tubers. The vector carrying this alternative version of the silencing cassette (pSIM1151) was used to produce 12 transformed lines. HPLC analyses demonstrated that tubers from most of the transformed lines contained the same asparagine levels as wild-type controls (Figure 4). Only three transformed lines produced tubers with less than one-half the amount of asparagine that accumulated in controls. This result confirms earlier studies, which indicated that tuber-specific and convergent transcription-mediated gene silencing is triggered more effectively by the combination of Gbss and Agp promoters than by two copies of the Gbss promoter (Yan *et al*., 2006). It can be concluded that silencing of the *StAs1* and *StAs2* genes caused a dramatic decrease in the accumulation of asparagine in tubers without compromising the yield of glasshouse-grown transgenic lines.

### Reduced asparagine formation in tubers limits heat-induced acrylamide formation in French fries

**Figure 5:**
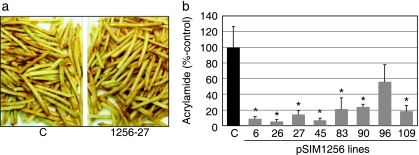
Acrylamide levels in French fries. (a) Visual appearance of fries from an untransformed control (C) and intragenic line 1256-27. (b) Acrylamide levels. Data are expressed as a percentage of the untransformed control value (1097 ± 258 ng g^−1^) and represent the mean of three measurements ± standard deviation. Asterisks indicate significant differences from controls (*P* < 0.05).

Potatoes are generally stored at temperatures that limit cold-induced sweetening (7–10 °C). For proof-of-concept experiments designed to determine the effect of low asparagine levels on acrylamide production, the storage temperatures were decreased to 4 °C. After a 1-month incubation period, tubers contained 8.8 ± 0.37 mg/g fresh weight (FW) of glucose, which is 12-fold higher than the glucose levels in freshly harvested tubers. Initially, the cold-stored tubers of eight intragenic lines (1256-6, 1256-26, 1256-27, 1256-45, 1256-83, 1256-90, 1256-96 and 1256-109) were blanched, cut, par-fried and finish-fried for 2 min 45 s for the production of lightly coloured French fries. HPLC analyses of these materials demonstrated that the control product contained 126 ng g^−1^ of acrylamide, whereas the presence of this undesirable Maillard reaction product was often below the detection limit of 20 ng g^−1^ in intragenic fries (data not shown). The finish-frying time was therefore extended to 4 min, and produced brown French fries for both untransformed controls and intragenic tubers (Figure 5a). As expected, the longer frying time resulted in the formation of more acrylamide in controls (1097 ng g^−1^), and low but detectable levels of this compound in intragenic fries (Figure 5b). Importantly, fries from lines 1256-26 and 1256-45 contained only approximately 5% of the acrylamide that accumulated in controls. The correlation coefficient between tuber asparagine levels and fry acrylamide levels was 0.9074, indicating that asparagine plays an important role in acrylamide formation.

The four intragenic lines 1256-27, 1256-83, 1256-90 and 1256-96 were evaluated by a team of professionally trained food scientists for various aspects of texture (crispness, mouthfeel, mealiness and texture variation) and taste (caramel taste, fresh fried taste and aroma). These assessments demonstrated that the above-described resemblance in the visual appearance of intragenic and control fries coincided with a similar texture and taste (Table 1). Collectively, our results demonstrated that a 3–18-fold decrease in the asparagine content of potato tubers resulted in a similar fold decrease in the amount of acrylamide accumulated during heat processing. These changes did not affect the tuber yield or French fry quality.

**Table 1:** Sensory evaluation of French fries prepared from both untransformed and intragenic Ranger Russet tubers stored for 1 month at 4 °C prior to processing. Tests were performed by a panel of eight professionally trained experts, who sampled French fries at the optimum time of 3 min. Data denote the average marking of six assessments ± standard deviation on a nine-point scale (9, excellent; 1, poor)

	Sensory characteristic	Control	1256-27	1256-83	1256-90	1256-96
Texture	Crispness	4.7 ± 0.5	4.8 ± 0.1	5.0 ± 0.1	4.5 ± 0.2	5.2 ± 0.1
	Mouthfeel	6.3 ± 0.3	5.8 ± 0.1	5.8 ± 0.3	3.8 ± 0.5	6.2 ± 0.2
	Mealiness	5.8 ± 0.3	6.2 ± 0.1	6.2 ± 0.2	5.2 ± 0.1	5.8 ± 0.2
	Variation	6.4 ± 0.3	6.2 ± 0.1	6.3 ± 0.3	6.3 ± 0.1	6.5 ± 0.3
Taste	Caramelized	6.6 ± 0.4	7.0 ± 0.0	6.3 ± 0.3	5.3 ± 0.4	5.8 ± 0.2
	Fresh-fried	2.9 ± 0.2	3.3 ± 0.4	3.0 ± 0.0	3.3 ± 0.4	3.3 ± 0.4
	Aroma	5.9 ± 0.7	6.0 ± 0.0	5.2 ± 0.2	5.3 ± 0.6	5.5 ± 0.4

### Production of low-acrylamide potato chips

**Figure 6:**
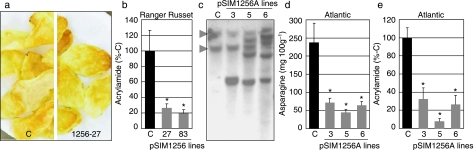
Potato chips from low-asparagine tubers. (a) Example of chips from tubers of untransformed Ranger Russet controls (C) and line 1256-27. (b) Acrylamide levels in chips. Data for chips from intragenic lines (grey bars) are shown as a percentage of the levels in chips from the untransformed controls (4318 ± 1137 ng g^−1^) (black bar). (c) Copy number of intragenic Atlantic lines 1256A-3, 1256A-5 and 1256A-6, as visualized with a probe containing fragments of both the *StAs1* and *StAs2* genes. Grey arrows indicate the positions of two endogenous hybridizing DNA bands. (d) Asparagine levels in lines 1256A-3, 1256A-5 and 1256A-6 (grey bars) compared with controls (black bar). (e) Acrylamide levels in processed material from intragenic Atlantic lines (grey bars) and controls (black bar). 100% = 840 ± 90 ng g^−1^.

The high surface-to-volume ratio of sliced tubers that are used for chip production promotes the rapid formation of acrylamide during heat processing (Matthäus *et al*., 2004). Therefore, the glucose concentration of cold-stored tubers was decreased (to 2.8 ± 0.12 mg/g FW) by incubation for 2 weeks at 15 °C. Potato chips obtained from reconditioned control tubers contained 4318 ± 1138 ng g^−1^ of acrylamide (Figure 6a,b), which is still approximately fourfold higher than that for French fries from cold-stored tubers (Figure 5b). Intragenic chips from lines 1256-27 and 1256-83 accumulated much lower levels of acrylamide (861 and 1153 ng g^−1^, respectively). However, these levels may still be sufficiently high to pose potential health issues. Thus, the impairment of asparagine biosynthesis is not always sufficient to guarantee low levels of acrylamide formation.

In an effort to optimize the decrease in acrylamide, the potato variety Atlantic, which contains much lower levels of glucose and fructose than does Ranger Russet (Webb *et al*., 1978), was transformed. Fifteen of 844 shoots regenerated from infected explants were shown by PCR-based genotyping to contain the *StAs1/2* gene silencing construct, whilst lacking backbone DNA, indicating a frequency for marker-free and backbone-free transformation of 1.8%. Plants of three intragenic lines that displayed reduced *StAs1* and *StAs2* gene expression levels, designated as 1256A-3, 1256A-5 and 1256A-6, were confirmed to contain at least one P-DNA insert (Figure 6c), and were grown in the glasshouse for tuber production. Harvested tubers accumulated 7.1%–18.4% of the asparagine that was produced in untransformed control tubers (Figure 6d). These fresh tubers were subsequently processed using standard procedures to produce potato chips. Figure 6e shows that control chips from Atlantic tubers contained 840 ng g^−1^ of acrylamide, which is about fivefold lower than the levels in chips from untransformed Ranger Russet tubers. Acrylamide levels were much lower in the processed material from intragenic lines, most notably line 1256A-5. This particular intragenic line contained only 8% of the acrylamide that was present in chips from untransformed Atlantic tubers (67 ng g^−1^), and may prove to be suitable for commercial production if none of the original agronomic characteristics of the Atlantic variety are lost as a consequence of the transformation process.

## Discussion

Simultaneous silencing of the *StAs1* and *StAs2* genes decreases the accumulation of free asparagine in potato tubers by up to 95%. Because asparagine plays an important role in nitrogen assimilation and storage (Lam *et al*., 2003; Lea *et al*., 2007), it was somewhat surprising that the targeted metabolic change did not affect the yield or shape of tubers from glasshouse-grown plants. To some extent, the intragenic tubers adjusted to the impaired asparagine biosynthesis by both accumulating more glutamine, which is the precursor of asparagine and has only a slightly lower nitrogen to carbon ratio, and increasing protein biosynthesis. It is also possible that tubers increased the formation of γ-aminobutyric acid, which contains about 11% of soluble nitrogen (Thompson *et al*., 1953). A full analysis of the extent to which intragenic tubers are capable of compensating for their lost ability to assimilate and store nitrogen in the form of asparagine will be carried out in future work. Regardless of the outcome of these studies, it was found that the tuber-specific silencing of the *StAs1* and *StAs2* genes did not affect the tuber yield of glasshouse-grown plants. This finding is in agreement with a previous study on transgenic potato plants down-regulated for the amino acid symporter StAap1 (Koch *et al*., 2003). Substantially decreased levels of free amino acids in the tubers of these transgenic plants were not associated with negative side-effects on leaf metabolism, tuber yield or phenotype.

In contrast with the tuber-specific silencing strategy employed in this work, it is predicted that strong constitutive silencing of the two related *As* genes would compromise plant growth and/or development. The proteins encoded by these genes play an important role in recapturing ammonium that is lost during photorespiration, a process that exceeds primary nitrogen assimilation by 10-fold (Keys *et al*., 1978). Although the enhanced assimilation of ammonium has been shown to increase photosynthesis (Fuentes *et al*., 2001) and accelerate growth (Oliveira *et al*., 2002), an impairment of this process may result in the accumulation of high and potentially toxic concentrations of ammonium (Gerendas *et al*., 1997; Wong *et al*., 2004). It should be mentioned that intragenic plants have not yet been exposed to the environmental stresses that are typical in the field. Such stresses include, for instance, drought, overwatering and infection, and are all known to enhance the formation of asparagine (Lea *et al*., 2007). The physiological significance of up-regulated asparagine biosynthesis is not yet fully understood, but it is possible that an inability to increase the concentrations of asparagine in tubers would, directly or indirectly, enhance stress sensitivity in the field.

Despite the efficacy of *StAs1/2* gene silencing, intragenic tubers still contained 5%–25% of the asparagine that accumulated in untransformed potatoes. Some of this amino acid may have been synthesized through either rudimentary StAs1 and/or StAs2 activity or transamination of 2-oxosuccinamic acid (Joy, 1988). A more substantial proportion was probably produced in leaves for transport through the phloem to the tubers (Fischer *et al*., 1998). Given the similar phenotypes of intragenic plants and their untransformed counterparts, it is unlikely that the silencing construct has a strong effect on the biosynthesis of asparagine in leaves. An additional argument for this hypothesis comes from the poor foliar activity of the promoters of the silencing construct (Visser *et al*., 1991; C. Richael, unpubl. data).

The significance of asparagine as an important determinant of the acrylamide potential of tubers was not noted in earlier studies evaluating the effect of genotype on acrylamide levels (Williams, 2005; Viklund *et al*., 2008). This discrepancy highlights the importance of other factors that influence the formation of acrylamide. Such factors include, for instance, glycine concentration (Bråthen *et al*., 2005), product matrix (Claeys *et al*., 2005), tuber moisture (Amrein *et al*., 2006), antioxidants (Zhang *et al*., 2007) and, most importantly, reducing sugars. The role played by each of these factors is variety dependent, which explains why asparagine concentration alone is not always a reliable predictor for the extent of acrylamide formation (Williams, 2005). Although the pSIM1256 Ranger Russet lines provided a suitable source of low-asparagine tubers for the production of French fries, they still yielded potato chips that contained relatively high levels of acrylamide. This result indicates that an impairment of asparagine biosynthesis is often necessary, but not always sufficient, to guarantee low levels of acrylamide formation during heat processing. The large amounts of reducing sugars that accumulate in cold-stored Ranger Russet tubers may accentuate reactions with compounds that represent alternative acrylamide precursors, such as glutamine (Mottram *et al*., 2002).

Our study demonstrated that low-asparagine Ranger Russet tubers that were cold stored at 4 °C yielded potato chips that were still relatively high in acrylamide. This finding indicates that the dramatic reductions in asparagine levels accomplished through *As1/2* gene silencing are not always sufficient to guarantee a low-acrylamide product. For chip production, it will still be necessary to either store tubers at higher temperatures (7–10 °C) or to apply typical ‘chip’ varieties, such as Atlantic. Alternatively, it may be possible to retransform a low-asparagine Ranger Russet variety with constructs for reduced cold-induced sweetening. Such constructs could be designed to down-regulate the expression of the genes involved in starch degradation (Rommens *et al*., 2006) or to express the maize (*Zea mays*) *Dof1* transcription factor gene (Yanagisawa *et al*., 2004) or the potato metabolic regulator SnRk1 (McKibbin *et al*., 2006).

The consumption of processed potato products contributes to approximately one-third of the average dietary intake of acrylamide (Boettcher *et al*., 2005). Thus, an eventual replacement of existing potatoes by low-asparagine varieties would lower the ingestion of acrylamide by approximately 30%. Even greater reductions could be accomplished by applying the described methods to wheat and coffee. In these cases, the *As* genes would have to be silenced in the endosperm.

In conclusion, our work has demonstrated that the heat-induced formation of acrylamide can be decreased by reducing the asparagine content in potato tubers. Preliminary data have indicated that this modification does not alter the normal agronomics of the crop. If supported by consumers, all-native fry products with very low levels of acrylamide could be offered as a new market choice within the next 5 years.

## Experimental procedures

### Database and statistical analyses

Gene expression levels were assessed by analysing expressed sequence tag data stored at the Gene Index Database, maintained by the Dana Farber Cancer Institute, Boston, MA, USA (http://www.danafarber.org). Amino acid motifs associated with the functional activity of enzymes were analysed by position-specific iterated and pattern-hit initiated blast (Schäffer *et al*., 2001). Databases searched using blastn and tblasts included those maintained by the National Center for Biotechnology Information, Bethesda, MD, USA (http://www.ncbi.nlm.nih.gov). The correlation coefficient between two variables was calculated according to Pearson (Gayen, 1951). Both the calculation of protein alignments and the visualization of cladograms were based on ClustalW (with penalties for opening and extending a gap set at 10.0 and 0.2, respectively). Protein structures were predicted using the phyre protein threading program version 2.0 (http://www.sbg.bio.ic.ac.uk/~phyre/). The *t*-test was used to study whether the means of two groups were statistically significantly different from each other.

### Plasmid construction

The DNA segment used to produce a multigene silencing construct contained 348-bp fragments of the *StAs1* gene (coordinates 318–665 of GenBank accession CK278037) and the *StAs2* gene (coordinates 314–661 of GenBank accession CK271149). The tuber-specific Gbss promoter represents coordinates 1138–1823 of GenBank accession X83220. The Agp promoter is identical to coordinates 2183–4407 of GenBank accession X96771. The basic P-DNA vector used has been described previously (Rommens *et al*., 2004).

### Plant transformation, genotyping and DNA gel blot analysis

Stock plants were maintained in Magenta boxes containing 40 mL half-strength M516 medium (PhytoTechnology, Shawnee Mission, KS, USA) with 3% sucrose and 2 g/L gelrite. Plants were transformed as described previously (Richael *et al*., 2008). Transformed plants were genotyped for the presence or absence of P-DNA, T-DNA and backbone DNA using a robust and reliable PCR method, explained elsewhere (Xin *et al*., 2003). The P-DNA copy number of intragenic lines was determined by isolating DNA using DNAzol reagent (Invitrogen, Carlsbad, CA, USA), transferring *Eco* RI digests to positively charged membranes (Roche Applied Science, Indianapolis, IN, USA) and hybridizing the membranes with a probe derived from the 0.8-kb DNA segment, which was employed to create the silencing construct and carried fragments from both the *StAs1* and *StAs2* genes, using the non-radioactive digoxigenin system (Roche Applied Science).

### RNA analysis

RNA was isolated from tubers using an RNAeasy Plant Mini Kit (No. 74904, Qiagen, Valencia, CA, USA), following the manufacturer's recommendations. cDNA was synthesized from 0.5 µg of total RNA in a 20-µL volume using an oligo dT primer and SuperscriptII RT (Invitrogen) at 42 °C in 1 h. One microlitre of resulting cDNA was used as template in the following PCR: 25-µL volume containing 20 mm 2-amino-2-(hydroxymethyl)-1,3-propanediol (Tris)-HCl (pH 8.4), 50 mm KCl, 1.5 mm MgCl_2_, 0.2 mm of each deoxynucleoside triphosphate (dNTP), 0.2 µm of each primer and 1.5 U Taq DNA polymerase (Invitrogen). The cycling protocol was as follows: 3 min at 95 °C, followed by 33 cycles of 20 s at 95 °C, 20 s at 57 °C and 80 s at 72 °C, with a final step of 2 min at 72 °C. Nine microlitres of the amplification products were analysed by electrophoresis and visualized by ethidium bromide on a 1% agarose gel. Semi-quantitative PCR for the *StAs2* gene was carried out with the pair of intron-spanning primers 5′-GGGATGCCATTGGCATTACAC and 5′-TGTACTGGCTCTGATACTGGTC, using Qiagen's one-step RT-PCR kit (catalogue number 210212), according to the manufacturer's recommendations. Intron-spanning primers for the internal control *actin* gene were 5′-AGTGGTCGTACCACCGGTATTGTG and 5′-ATGATCAGTGAGGTCACGACCTGC. Non-radioactive digoxigenin RNA gel blot hybridization was performed according to the manufacturer's recommendations (Roche Applied Science). A labelled 1.1-kb probe for the *StAs2* gene was derived from a gene fragment amplified with the primer pair 5′-CTTGCTCATCAACGATTGGCAATAG and 5′-AGGTCGGATCATTTTCCATTCTG. The primers used to produce a 1.1-kb probe for the *StAs1* gene were 5′-GGTTGATGACTGATGTCCCCTTTG and 5′-TAGTTAGCTCCTTATTGTGAGCTC.

### Tuber production, characterization and processing

Plants were grown for 3 months in 7.6 L pots in a glasshouse that was controlled for temperature (18 °C minimum/27 °C maximum) and light (16-h photoperiod with an intensity of approximately 1500 µmol/m^2^/s). The specific gravities of the harvested tubers were determined by dividing the weight in air by the weight in water (Kleinkopf *et al*., 1987). For French fry production, the tubers were washed, blanched for 8 min at 74 °C, cut into shoestring strips, dipped in a 1% sodium acid pyrophosphate solution at 71 °C, dried at the same temperature, fried at 200 °C for 45 s and frozen for 20 min at –26 °C, shaking the tray two to three times in the first 6 min. The processed fries were then finish-fried at 168 °C for either 2 min 45 s (light colour) or 4 min (dark colour). Potato chips were produced by slicing fresh tubers to 1.0-mm thickness using an industrial slicer; after air drying, they were fried for 11 min at 170 °C.

### Assays for glucose, amino acids and total protein content

A glucose oxidase/peroxidase reagent (Megazyme, Bray, Co. Wicklow, Ireland) was used to determine the glucose levels of cold-stored tubers. For amino acid analyses, tuber samples were extracted and analysed using an automated amino acid analyser according to the official methods of analysis of the Association of Official Analytical Chemists (AOAC) (http://www.aoac.org/omarev1/982-30.pdf). The total protein content was determined by grinding a transverse slice of approximately 0.5 cm across the middle of the tuber in liquid nitrogen with a mortar. A homogenate containing 250 mg of the ground powder in 0.5 mL of extraction buffer [25 mm Tris-acetate pH 8.5, 0.5 m NaCl, 5 mm phenylmethylsulphonylfluoride (PMSF)] was centrifuged at 12 000 ***g*** for 10 min. The protein concentration in the supernatant was then measured using the Bradford protein assay kit (Bio-Rad, Hercules, CA, USA).

### Assessment of fry colour and acrylamide levels

Colour was assessed using an E30-FP Agtron Process Analyser (Agtron, Reno, NV, USA), whereby a lighter colour is reflected by a higher number and values above 40 are generally considered to be acceptable. French fries were ground to a fine powder in liquid nitrogen; the powder was shipped on dry ice to Covance Laboratories (Madison, WI, USA), where a combination of liquid chromatography and mass spectrometry was used to detect acrylamide at concentrations as low as 20 p.p.b., according to a method developed by the United States Food and Drug Administration (http://www.cfsan.fda.gov/~dms/acrylami.html), adapted from an earlier method (Schuster, 1988).
